# Establishing undergraduate public health education: process, challenges, and achievements in a case study in Israel

**DOI:** 10.1186/s40985-017-0057-4

**Published:** 2017-04-03

**Authors:** Osnat Bashkin, Theodore Herzl Tulchinsky

**Affiliations:** 1 Department of Public Health, School of Health Professions, Ashkelon Academic College, Ashkelon, Israel; 2 School of Health Professions, Ashkelon Academic College, Ashkelon, Israel

**Keywords:** Undergraduate public health, Education, Curricula, Democratization of public health career entry

## Abstract

**Background:**

In this paper, we describe the development process of the first undergraduate public health baccalaureate program, in the Ashkelon Academic College in Israel. Expansion of degree-granting colleges in Israel is part of the democratization of higher education providing access to and increasing educational opportunities for groups underrepresented in universities. The main objectives of the program at Ashkelon Academic College have been to open accessible and affordable career opportunities for current workers in the health system and for new entries to health careers for academic advancement in a peripheral and relatively poor region of the country.

**Case Presentation:**

The program focuses on well-established and literature-based learning goals of public health education but also includes basic medical sciences, incorporation of arts and sciences into public health, development of critical thinking and quantitative skills, experiential field learning, and integrative learning for facing global health challenges. The curricula of the program is composed of seven modules including introductory courses, methodology courses, health organization courses, epidemiology courses, courses related to core content of public health, elective courses and practicum. The first class will graduate in 2017; this will allow for final approval of the Council of Higher Education of Israel and possible revision of curriculum. A second BA program is now seeking approval in Israel and cooperation with post-graduate schools of public health is developing.

**Conclusion﻿s﻿:**

Our program is in keeping with trends in Europe and the USA to broaden public health education, to reduce inequality of career opportunity, to expand the workforce, and to promote public health.

## Background

In 1915, the Welch-Rose Report promoted the concept of formal public health education at universities in the USA. The few schools initially established slowly grew to 12 schools by 1960, increasing to 46 accredited schools in 2011 [1] and to 59 accredited schools of public health in 2017 [2]. The US Institute of Medicine (IOM) report published in 2003: *Who Will Keep the Public Healthy? Educating Public Health Professionals for the 21st Century* [3] stated, “A public health professional is a person educated in public health or a related discipline who is employed to improve health through a population focus. Nearly all public health professionals encompassed by this definition have earned at least a baccalaureate degree.” The report also recommended that students from undergraduate programs should be encouraged to advance in public health education [4]. These recommendations were supported by the Consensus Conference on Undergraduate Public Health Education sponsored by the Association of Schools of Public Health (ASPH) and others [5]. The recommendation included accessibility to a common core curriculum in public health at all undergraduate institutions of the arts and sciences [6].

In Europe, following the Bologna Declaration of 1999 [7] calling for a reorganization of higher education in Europe, the Association of Schools of Public Health in the European Region (ASPHER) established a Working Group on Undergraduate Programmes in Public Health and conducted a survey of undergraduate programs in schools of public health across the European Region [8]. Revision of public health education in Europe, as elsewhere, requires review of curricula and competencies for undergraduate and graduate levels along with international accreditation systems to meet the qualitative and quantitative needs of a strong public health workforce [9].

Despite the growth in public health undergraduate education programs in the USA and Europe over the past 15 years and the growth of interest in public health, there are still major challenges. There is still a larger policy and resource allocation focus of government attention to medical care as compared to public health, which limits the capacity of public health [10] to face contemporary and future challenges of health promotion and disease prevention. In low- and medium-income regions or countries, undergraduate public health education is especially crucial for training of the public health workforce to address longstanding and emerging public health problems and the real needs of public health programs in developed as well as developing countries [11, 12].

A growing part in the development of public health workforce in Europe, as in the USA and in other countries, is undergraduate level education. In the Netherlands, Maastricht University since 2006 [13], and in Lithuania, Kaunas and Vilnius Universities since 1994 [14], are longstanding pioneers in bachelor training in public health as well as master’s and PhD levels. East London University in the UK joined their ranks in 2009 with BSc and MSc public health programs [15]. In this paper, we describe the development process of the first undergraduate public health baccalaureate program in Israel, discussing its challenges and achievements.

## Case ﻿Presentation

### The higher education system in Israel

**Fig. 1:**
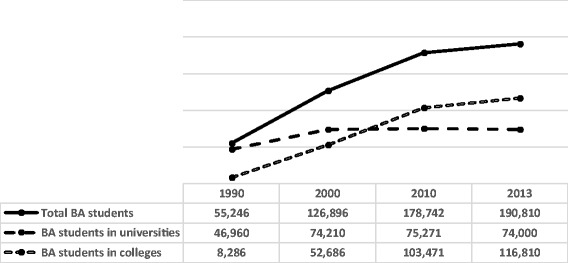
Trends in the number of undergraduate students in colleges and universities in Israel over the period 1990–2013. Source: [19]

The main aim of the higher education system in Israel is to provide rigorous academic standards and universal access for career opportunities and adequate workforce in many fields. Higher education in Israel has more than tripled since the 1990s, with the number of undergraduate students increasing from 56,000 in 1991 to 195,620 in 2016 [16, 17]. Much of this growth is due to the establishment of new colleges, which concentrate mainly on undergraduate programs in peripheral regions rather than on research or advanced education programs in the main cities [17]. The Council for Higher Education of Israel (CHE) promoted expansion and accreditation of undergraduate education in academic colleges in response to the growth of the population, and the public and labor market demand for higher education [18]. The total number of colleges grew from 6 in 1989–1990, to 37 in 2013–2014 as seen in Fig. 1 [19].

In the decade between 1990 and 2000, the number of students studying for baccalaureate degrees in colleges and universities in Israel grew by 130%. In this decade, the number of students studying for baccalaureate degrees in colleges grew by 500% whereas the number of students studying for baccalaureate degrees in universities grew by only 50% [19].

The academic colleges in Israel create greater equality in opportunities for higher education for less advantaged populations. They provide accessibility to higher education to residents of the geographical and social periphery, as well as to minorities such as Arabs, Bedouins, and ultra-orthodox Jews. These colleges provide access to residents of underserved regions at lesser total costs to students associated with travel or residence in the main university centers, while allowing students to simultaneously work and conduct normal family lives. Colleges reduce migration of populations to the central cities of Israel. In addition, colleges promote multi-culturalism and diversity by enrolling students from all socio-demographic statuses. Colleges also provide educational upgrade opportunities for people working in social and health services but lacking a baccalaureate degree needed to advance in their careers [20].

The undergraduate public health program described in this paper was established in the Ashkelon Academic College, located in the southern part of Israel. There are a variety of academic tracks for BA, as well as BSc and new master’s programs in the college, including tracks in Social Sciences, Humanities, Economics, Criminology, Computer Sciences, Tourism, and the new School of Health Sciences.

## The purpose of public health undergraduate education in Israel

The healthcare system in Israel includes a national health insurance program providing universal coverage to all citizens through four health service funds under the National Health Insurance Law of 1995. Striking progress has been made in recent decades with improving health quality measures, such as life expectancy, yet there are still major challenges for the public healthcare system in Israel, including the increase in rates of diabetes, obesity, chronic diseases related to aging of the population, inequalities, and other issues. More challenges have emerged such as environmental changes, public health implications of violence, and new worldwide pandemics of infectious and other disorders [12].

These challenges are very relevant to the public health system in Israel. The efficiency of the public healthcare system relies on an infrastructure of well-trained multidisciplinary professionals who are capably involved with designing and implementing public health promotion programs to meet these challenges. As of 2014, Israel had four university graduate programs in public health with MPH and PhD degrees. The growing need for public health workers in the Israeli healthcare system, especially in the peripheral south of the country, led us to propose and develop the first Israeli undergraduate program in public health. The main purposes of the program were to create a basis for new health professionals for different areas of public health and to provide current health workers lacking higher education the opportunity to acquire a formal, academic degree that would allow them to advance in their careers.

## Development process of the public health undergraduate program

**Fig. 2:**
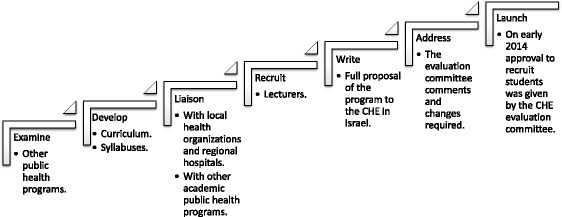
Framework of the public health program development process

The development process began in 2010 with a proposal to the president of Ashkelon College to establish a BA program in public health. The College adopted this as part of its 5-year development plan. After extensive preparation, and proposal submission and revision, in 2011, the CHE of Israel approved the establishment of a school of health sciences in the Ashkelon Academic College. As part of the process, the College decided to develop several health profession baccalaureate programs in public health, nursing, and nutrition. Figure 2 outlines the framework of the public health program development process.

We started by examining other public health programs in different countries around the world, such as those in Europe and the USA where there are many established undergraduate public health programs. In addition, we examined the specific professional needs of the healthcare system in Israel including the need for a well-qualified, undergraduate-level educated, public health workforce, to help cope with emerging public health problems and promote public health. In the Israeli healthcare system, public health nurses, whose job descriptions are focused on maternal and child health, do not necessarily possess the appropriate knowledge of other topics of public health importance, which are not included in the nurses’ daily work. This situation resulted in a heavy workload for public health nurses and neglect by the Ministry of many important public health problems.

The next stage was to develop the curriculum of the program and describe the structure of the courses to be included. We recognized the importance of establishing liaison with the local regional hospital and public health offices. Therefore, we met with health professionals, health service executive officers, and hospital managers to discuss the program and identify cooperation opportunities. At the same time, we recruited professionals and managers from the health system as lecturers willing to commit to become part of the academic staff of the program. During this period, we wrote the complete detailed proposal of the new program for the CHE of Israel. The proposal included the specific purpose of the program, the lecturers’ curricula vitae (CVs), course syllabuses, the colleges facilities required for the programs (for example labs and library), references, and other academic requirements.Some of the syllabuses were new and had never before been taught in Israel or in other public health programs.

The CHE set up an evaluation committee, in order to examine and evaluate the new program. During a period of almost 2 years, changes required from the committee were addressed and resubmitted for re-evaluation. Throughout this evaluation process, the program was altered to meet CHE requirements going beyond the usual curriculum requirements of bachelor programs abroad and refined until approval was granted to proceed and recruit students in early 2014.

## Curriculum content

**Fig. 3:**
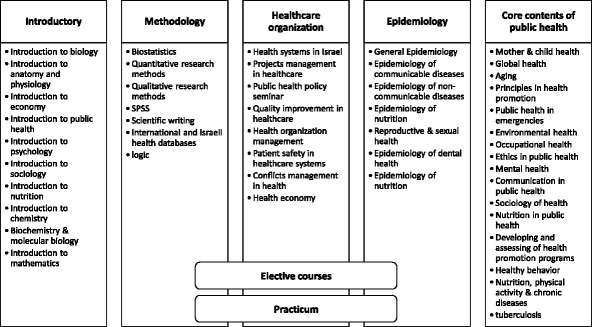
Curriculum of the public health program in the Ashkelon Academic College

There was a large variation between public health undergraduate programs we examined in curricular contents, teaching methods, and resources. Nevertheless, there were common core and cross-cutting subjects taught in most of the undergraduate programs we accessed. In developing the curricula, we focused on the main well-established learning objectives of public health education: integrating the contribution of arts and sciences disciplines into public health, developing critical thinking and quantitative literacy skills, acquiring personal and social responsibility through incorporation of experiential learning, and providing integrative learning for global health challenges [21]. At the behest of the College and of CHE, we included basic biological subjects, mathematics and other core courses thought necessary for new entries in the health field. Several main study modules derived from our new public health program purposes. Curricular contents of each module are presented in Fig. 3.

The program’s curriculum contains seven modules. The first module contains a range of introductory courses related to biological and social sciences, public health, and other health professions; many of these courses address the first learning goal of integrating arts and sciences disciplines into public health. The second module contains methodological skills including biostatistics, qualitative and quantitative research methods, and writing skills. This module addresses the second learning goal of critical thinking and quantitative literacy skills. The third module contains courses related to the healthcare organization including management, quality, safety, economy, and policy. Most of the courses in this module address the learning goal of providing integrative learning for national and global health challenges. The fourth module contains a wide range of courses in core topics of public health, which addresses the learning goal of providing integrative learning for national and global health challenges. The fifth module contains epidemiology methodology and epidemiology of infectious and non-communicable diseases. Courses in this module address the second learning goal of critical thinking and quantitative literacy skills. The sixth module called “elective courses” allows the students to participate in four courses from other BA programs in the College, aimed to enrich their knowledge in areas not necessarily related directly to health.

The seventh and final module contains a practicum in the third year of the program. In the practicum, the students are required to conduct a research project on a public health topic, to design and develop a health promotion intervention, or to conduct a survey examining a public health issue. Academic staff members guide our students through this process, along with an external professional mentor working in a local health organization where the study is carried out. This module aims at addressing the learning goal of acquiring personal and social responsibility by incorporating experiential learning.

The introductory level courses and the methodological courses provide the students with skills studied as core topics of public health and those required to perform research in these topics. The practicum promotes experiential learning; it provides the students with practical skills and with community-based research skills.

## Discussio﻿n

### Barriers, achievements and ongoing efforts

Undergraduate public health education being a new concept in Israel, we faced several barriers throughout the process. As mentioned, until now, there were only MPH and PhD university programs in public health in Israel. We were pioneering the development of the first baccalaureate degree in public health in Israel. In addition, we developed the program for a college located in the peripheral southern region of the country. This region faces a lack of accessible opportunities for higher education and an unstable security status. Ashkelon is located only 14.5 km from Gaza and faces frequent threats, actual rocket bombardment and periodic wars one of which while we were recruiting our initial cadre of students. Further, the closest university is 60 km away. The socioeconomic status of the local population is low, and paying for tuition and other costs in university centers far from home is out of reach for most people. Moreover, there were no defined positions in the health system for bachelor degree graduates in public health and potential candidates wanted to know what they could do after they finished studying. Public health itself was not a widely known topic among the local population. We overcame some of the challenges mainly through cooperation with local health organizations and with public health professionals from postgraduate programs in the universities. The first class included 14 students; the second class had a disappointing 10 students; but the third class, beginning studies in 2016, enrolled 20 students.

Parallel to the challenges that we are still facing, there were several major achievements during the process. We attracted well-qualified teachers including young faculty with PhDs, MDs, and MPHs, and older, experienced teachers retired from other academic centers. All were committed and cooperative. We established a multidisciplinary, community-partnership-based education in public health including a diverse curriculum, and a final year practicum, which is carried out in local health organizations. This practicum in community health services is a significant part of the curriculum and is important for the students, as well as for the community of the south. We established strong connections with the Ashkelon regional hospital (Barzilai), which allowed a day off work for its staff taking our courses and provided financial support to cover tuition costs. In addition, the director of the regional hospital conducted a course in health administration for the students in the program. We established strong relations with public health organizations and university postgraduate degree centers, in Ben-Gurion University and the Hebrew University. The International MPH program class of 2015–2016 from the Braun School of Public Health visited our program along with the director of the school and director of the IMPH program. Some of our current students were very much influenced by meeting IMPH students and are planning to apply for MPH and PhD programs. We have full cooperation with related programs, such as Nutrition and Nursing, which are part of the same School of Health Sciences at the Ashkelon Academic College.

Student and lecturer motivation and satisfaction are high. Students report interesting courses and warm and supportive relationships with lecturers. Meanwhile, lecturers detail interesting classes and fruitful discussions with students. Some of our students are diploma level workers in the health field such as diploma level nurses, dental hygienists, medical technologists or administrative workers in local or regional hospitals or primary care clinics. The program provides them with education opportunities for career advancement, and an academic degree is essential to advance. We also provide opportunity for entry into the health field for young students who could not afford to go to university centers in central Israel. We are currently concluding arrangements with the Hebrew University Dental Faculty for a joint program for Dental Hygiene students. Students will acquire a BA in public health and a diploma in dental hygiene, and we hope to do the same with Medical Technology diploma students.

Lessons learned in this first experience of our program include the following:

1. Competency of students
  The students in the program described included people with diplomas in health fields such as dental hygienists, nurses, medical technicians, and administrative personnel from health providers including hospitals and clinics. Some had graduated with diplomas many years ago and had practiced and worked in their professions while raising families. Their enthusiasm and performance in class and in examinations was unexpectedly exceptionally high. New entry young students also performed beyond expectations. The students’ identification and interaction between themselves and with faculty was continuously warm and active, with fruitful interrelationships.
2. Involvement of faculty
  Motivation of faculty members recruited from well-established universities and major health providers in the region included strong willingness to help in development of an underserved and relatively neglected region of the country. This was translated into a high commitment to the program and supportive relationships with students. There were several withdrawals from personal reasons such as work overload, but the core group of academic staff was sustained, and competent replacements were found.
3. Curriculum
  Some differences in curriculum from other programs abroad were required by the CHE such as core courses in basic sciences including biological sciences, chemistry, mathematics, logic and others, on the assumption that these were necessary for new entries into the health field. These subjects were not included in most of the MPH programs. This caused the curriculum to go beyond the common requirements of BA programs in the College and abroad. This difference reflects the CHE assumption that the Israeli public health workforce requires strong preparation in basic sciences. We believe that there is some justification for this requirement so that public health workers should possess basic understanding of biological as well as epidemiologic and social sciences, although we may make changes in the curriculum balance in the future. During the initial period of approval, we were not allowed to make changes in curriculum until final approval to issue the BA to the first group of graduates. Following final approval with the first graduating class, the academic committee of the program at the Ashkelon Academic College will review the curriculum with the view to its compatibility with MPH programs and BA programs abroad. Final review of the curriculum is now in process.
4. Practicum
  We have found that the practicum is a significant part of the curriculum. Its length is a full academic year in the final year of the program. The students may participate in the practicum only after completion of all former studies in the first 2 years including introductory and core courses. This provides them the tools to undertake a research experience in healthcare facilities such as the regional hospital or community clinics. The students work in pairs under supervision of a faculty member as well as supervisors from the facility in which the research is conducted. The students are required to write periodic reports and present their final project in a local conference. The practicum research subjects vary from epidemiologic research to health promotion interventions and health surveys. The practicum provides hands on experience, which we believe to be a value in expanding competency for future career development and provide the students with experiential field learning.
5. Academization
  The new program provides the students with critical thinking, quantitative as well as qualitative skills, experiential field learning, and integrative learning skills for facing national health challenges. The program provides local health workers in the peripheral region of the country, the empowerment to advance. Students in the new program, whether they possessed some experience in the health field or whether they were young people who wished to work in the health field, acquired competencies to think “out of the box,” to professionally interpret data, to research and write academic papers, to conduct health surveys and health promotion interventions, and to deal with health challenges.
6. Next steps for graduates
  Because the BA in Public Health program was new in Israel, we were unable to guarantee defined positions in the health sector for our graduates. However, we assumed that the graduates would be strong applicants for positions at entry levels in various services in the health field. In addition, people with work experience would be eligible for career advancement including salary increases based on acquiring a BA. Currently, in the third year of the program, many of the students have expressed intentions to apply for MPH programs in one of the four schools of public health in Israel. We are keeping contact with public health schools in universities with regard to enrolment of our graduates for MPH and PhD studies. This factor will be taken into account in curriculum review and modification of the structure of the program after final approval from CHE has been granted. We have recently achieved formal agreements with the four schools of public health in Israel to a joint project that includes harmonization of curriculum of public health programs.

Specific job creation for our graduates has not yet occurred, but we have been encouraged by support of the Ministry of Health, and health providers in the region that our graduates may be recruited to suitable entry-level positions in public health, research, health providers, and possibly in health industry. Our program offers entry or advancement to people who wish to enter or are already working in the health field.

## Conclusions

Our program is still new, and we still face major challenges, in student recruitment, employment following graduation, and career development with continuation to master’s and perhaps PhD levels for which some students are showing aptitude and motivation. More than half of our current students studying who are now in their final year of the program are planning to apply for master’s and hope to go onto PhD programs in existing public health schools in Israel. Moreover, our teachers and students are involved in a new approach providing a stimulating and warm relationship, in a pioneering new enterprise in Israel. Our new bachelor level training in public health is consistent with similar education programs in the USA and in Europe. The University of Haifa in Israel has already applied to the CHE and is in the process of developing an undergraduate program in the field of health promotion. We believe undergraduate education in public health can be an important model for public health personnel, for developing and developed countries.

## Abbreviations

ASPH: Association of Schools of Public Health
ASPHER: Association of Schools of Public Health in the European Region
CHE: Council for Higher Education of Israel
IMPH: International Master of Public Health
IOM: US Institute of Medicine
MPH: Master of Public Health
